# Do consumers care about substances of very high concern in articles?

**DOI:** 10.1186/s12302-018-0153-1

**Published:** 2018-08-21

**Authors:** Sabrina Hartmann, Ursula Klaschka

**Affiliations:** 1 University Ulm, Albert-Einstein-Allee 11, 89081 Ulm, Germany; 2 University of Applied Sciences Ulm, Prittwitzstr. 10, 89075 Ulm, Germany

**Keywords:** Articles, Consumers’ awareness, Hazard communication, REACH Regulation Art. 33(2), Substances of very high concern (SVHC), ‘Right to know’, Risk communication

## Abstract

**Background:**

Consumers have the right to inquire whether a consumer article contains substances of very high concern (‘SVHC right to know’). This communication tool is designed to stimulate suppliers to substitute such ingredients. A survey among 1321 consumers with high motivation and interest in harmful substances in everyday products was conducted to understand the acceptance of this ‘right to know’ among consumers.

**Results:**

Only one out of seven survey participants stated to be well informed about the ‘SVHC right to know’ with nearly all of them having good self-reported chemical knowledge. Three quarters of the participants who are not working with chemicals or REACH at their workplace have never heard about the ‘SVHC right to know’. Every second participant declared their interest to search for more information about an SVHC in a certain article, but, in fact, not more than 4% of all participants inquired for SVHCs with various methods. Only 1% would buy an SVHC-containing article with no strings attached. While detailed comments by some survey participants showed a high level of understanding of the issue, many respondents were not sure what the SVHC information means for their daily life. They declared that they would inform themselves, reduce the use of the article with SVHCs, circulate this information, or throw such an article into the garbage. Most study participants suggested improvements of the ‘SVHC right to know’. The preferred suggestions were a ban of SVHCs, easily understandable information on the packaging, full ingredient declaration on the articles, or no need to inquire for every single item, while smartphone applications for SVHC requests were the least popular suggestion in all age groups.

**Conclusions:**

Various reasons could be identified why most consumers—even these motivated and interested ones—do not use the ‘SVHC right to know’. This allowed developing recommendations for improving the effectiveness of this communication instrument on the way to the gradual elimination of SVHCs in consumer articles.

**Electronic supplementary material:**

The online version of this article (10.1186/s12302-018-0153-1) contains supplementary material, which is available to authorized users.

## Background

The European chemicals’ regulation REACH 1907/2006/EC (REACH Regulation) [1] is an important driver on the way to the ambitious goal of a ‘non-toxic environment’: the seventh environment action programme mandates the European Commission to develop by 2018 “a Union strategy for a non-toxic environment that is conducive to innovation and the development of sustainable substitutes including non-chemical solutions” [2, 3]. One element for achieving this goal is the provision set-up for substances of very high concern (SVHC) in the REACH Regulation. According to Art. 33(2) [1], consumers have the right to receive information from the suppliers of an article about the presence of any SVHC in the article, its subassemblies, or its packaging above a threshold of 0.1% (weight/weight) upon request. Information shall be provided within a time period of 45 days free of charge. SVHCs are chemicals which are carcinogenic, mutagenic, and toxic for reproduction or very critical for the environment [because they are persistent, bioaccumulative, and toxic (PBT) or very persistent and very bioaccumulative (vPvB)] or which cause concern for other reasons such as endocrine disruptors ([1] Art. 57). They are listed in the so-called candidate list [4] which is updated twice a year (last update 01/2018 with a total of 181 entries). These substances are candidates for inclusion in Annex XIV, which is the list of substances subject to authorization (Art. 56, 58) [1]. Authorization means that a manufacturer, importer, or downstream user shall not place a substance on the market, if that substance is included in Annex XIV, unless the special use has been authorized according to the provisions laid down in Art. 60–64 or the substance is exempted from authorization (Art. 56).

The ‘SVHC right to know’ is a key element on the way to minimize adverse effects for the consumer and the environment caused by harmful chemicals. It should increase transparency and awareness on SVHCs in consumer articles, help interested consumers to make informed purchasing decisions, and to ensure a safe use of articles, and it should contribute to a reduced discharge of SVHCs into the environment and support the efforts of producers to substitute SVHCs by less harmful chemicals or non-chemical solutions. There are companies that take great care and answer the requests to the best of their knowledge. However, there are various studies describing that this communication tool is not working as well as intended. The problems identified are for example, that not all suppliers are aware of their obligation, that the quality of the answers often is not satisfactory, or that consumers receive no answers at all for articles which contain SVHCs above the threshold [5–8]. Some competent authorities made efforts to support suppliers with their duty to inform [7, 9], whereas the role of the consumers in this risk communication has been neglected so far. This could be one reason why the ‘SVHC right to know’ has still not become very popular, although it has been in force for more than 10 years now.

In 2016, we conducted an online survey which investigated the awareness of 1321 consumers on harmful substances in everyday items [10]. Participants were not representative for the general population, but represented the intended ‘best-case’ scenario, as nearly all of them indicated that they were interested in chemicals in everyday products and the majority had a higher education level and good self-reported chemical expertise (Additional file 1: Figure S1). Thus, the motivation to observe and the capability to comprehend risk communication elements in this group are assumed to be higher than in the average population. The underlying hypothesis for the approach used was that motivated consumers, who take a high interest in chemical ingredients in everyday products and who have good knowledge of the matter, would use the ‘SVHC right to know’ more frequently than the average consumer. Our analysis of the first part of the survey [10] had shown that motivation and knowledge in chemistry helped, but did not exclude misconceptions about harmful substances in products. The present analysis covers the second part of this survey and analyses answers given to questions about the ‘SVHC right to know’. It aims at finding out whether this communication instrument is useful for reducing the risk for man and the environment to the unavoidable minimum. It examines consumers’ acceptance of the ‘SVHC right to know’ and searched for answers to the following questions: Which consumers know their ‘right to know’? How many are using it? What are their expectations and evaluations?

## Methods

### Approach and data analysis

The online survey was executed using the cloud-based software provided by the service company SurveyMonkey (http://www.SurveyMonkey.com) as described in [10]. It was accessible in the internet between 13th September and 31st October 2016. Participants responded to a maximum of 38 questions, with various branch points (Additional file 2: Figure S2). Answers given to Questions 1–14 are analysed in [10], and also the demographic data are presented and discussed there. The questionnaire was distributed in German language. The English text of the questionnaire (Questions 15–38) is in Appeρndix, the original German version in the Additional file 3). All information collected was self-reported by the participants. Several questions offered the option to add free text. These comments were analysed qualitatively.

Data received from the software provider comprised the numbers of responses and the date of the participants’ online access. The demographic information collected did not allow for the identification of any participant of the survey. It must be noted that originally 1321 persons had started the questionnaire, while only 1030 arrived at the last question and indicated demographic information needed for the statistical analysis of the answers (Additional file 2: Figure S2). Therefore, only the answers of the participants who reached the end of the survey were evaluated with respect to the demographic variables. The correlations between the responses and the demographic details of the participants were calculated using the software application ‘matrix laboratory’ (MATLAB) (https://www.mathworks.com). The age group below 20 years with only 25 respondents was too small for separate statistical analysis. Pearson’s chi-squared test was used to detect correlations for data sets containing nominally scaled variables. *p* values below 0.05 were considered significant.

### Study limitations

In this survey, the sampling of the study participants was not random, but represented a ‘best-case’ selection; however, the self-reported demographic data were not verified and the age classes of the participants were not equally distributed [10]. The demographic question concerning the educational qualification level offered not more than six potential answers so as to allow the authors a pragmatic analysis of this demographic factor. The study participants who indicated to be members of environmental or consumer organizations did not have to name the organization. Study participants could report whether they considered themselves or family members to have a chemical intolerance according to their own criteria, and it was not differentiated between allergic skin reactions, food intolerance, respiratory diseases, or other health problems. The answers given by the study participants were not verified by other means. Misconceptions leading to wrong answers and unintentionally given incorrect answers which are in accordance with the general expectations cannot be ruled out.

## Results

### Consumers’ knowledge and expectations of the REACH Regulation

Nearly all participants (98%) indicated in the first question of the survey that they were interested in chemicals which are harmful for human health or the environment and used in everyday products (such as, for example, articles like electronic devices or toys, mixtures like cosmetic products or pharmaceuticals, or food products) [10]. These persons were forwarded to the subsequent questions (see Appendix). There were several bifurcation points as illustrated in Additional file 2: Figure S2. The analysis of the answers to Questions 1–14 was published in [10].

Survey participants were asked in Question 15 whether they knew the European Chemical Regulation REACH [1]. Persons who affirmed this question (637 individuals) could indicate their personal opinion about the REACH Regulation in Question 16.

Two-fifths (39.6%) of the participants indicated that they had never heard about the REACH Regulation before, while a third (33.2%) had heard it before and around a quarter (27.1%) of the participants indicated that they had a good knowledge about it. Nearly all participants (92%) who are working with chemicals or REACH at their workplace knew the REACH Regulation well or have heard about it (*χ*^2^ = 438.5, *p* < 0.0001). Nearly three out of four (71.8%) persons with very good knowledge in chemistry indicated to know the REACH Regulation well (*χ*^2^ = 359.8, *p* < 0.0001). Four out of ten (42.4%) participants who are not working with chemicals or REACH at their workplace knew the REACH Regulation. The age group between 30 and 39 was informed best (34.8% of this age group know REACH well), with decreasing percentages towards the younger age groups as well as towards the older age groups (*χ*^2^ = 40.4, *p* < 0.0001). More than every second person above 70 (51.9%) had never heard about the REACH Regulation. Two-thirds of persons with a university degree or Ph.D. (66.4%), every other student or trainee (48.6%) and two-fifths of persons with completed vocational training or apprenticeship (41.0%) and foremen resp. business administrators (41.5%) indicated to have heard about the REACH Regulation or to know it well (*χ*^2^ = 74.6, *p* < 0.0001).

**Fig. 1:**
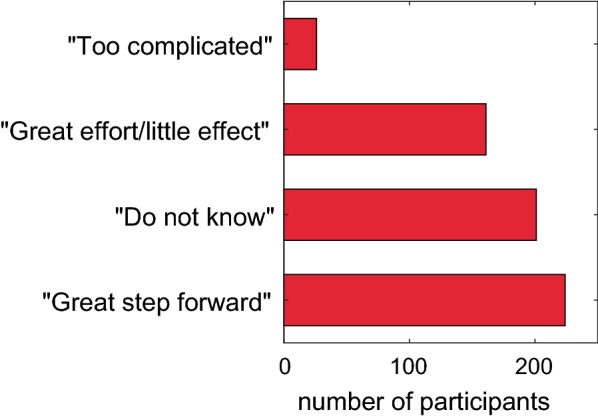
Subjective assessments of the REACH Regulation (answers to Question 16)

More than a third (35.4%) of the respondents considered the REACH Regulation as a great step forward leading to a better handling of chemicals (Fig. 1). Every second person who is working with chemicals or REACH at their workplace (49.5%, *χ*^2^ = 50.1, *p* < 0.0001) and who has very good knowledge of chemistry (48.7%, *χ*^2^ = 19.8, *p* < 0.0001) was of this opinion, while only 22.0% of the participants who are not working with chemicals or REACH at their workplace (*χ*^2^ = 50.1, *p* < 0.0001) considered REACH as a great step forward. More than every second participant (56.0%) who indicated to know the REACH Regulation well (according to the answer to Question 15) considered the REACH Regulation as a great step forward, while a third of this group (33.1%) thought that it is a great effort with a little effect. In total, a quarter (25.4%) of the respondents considered the REACH Regulation as a great effort with a little effect. More than a third (36%) of persons with very good knowledge in chemistry gave this answer (*χ*^2^ = 17.9, *p* < 0.0002). A third (31.8%) of the respondents stated that they did not know. This answer was given by every other person (51.9%) who is not working with chemicals or REACH at the workplace (*χ*^2^ = 89.1, *p* < 0.0001), as well as every other person (50.7%) with no self-reported knowledge in chemistry (*χ*^2^ = 55.9, *p* < 0.0001). Only 4.3% of the participants declared that the REACH Regulation was too complicated for them.

The strong commitment of the participants in answering the survey was reflected in the free-text annotations made to Question 16. Nearly all the 46 comments were indicative of a profound knowledge of the REACH Regulation. Twenty participants wrote that the REACH Regulation would be positive in general, but with restrictions (e.g., ‘it would not be sufficient’ or ‘the great efforts would lead to little result’). Eight persons criticized that the controls and sanctions for the ‘SVHC right to know’, for the registration dossiers, and for imported articles were not sufficient. Some of them also stated that the ECHA and the manufacturers had too much power. Five comments addressed the major expenses and great efforts for small and medium companies and for suppliers of complex products. Three participants wrote that economic interests would outweigh consumer and environmental issues, and the instrument of socio-economic analysis in the REACH Regulation was given as example. Three comments asked for a better information transfer to the public. Two participants were against the REACH Regulation because of its demand for animal testing. One person stated that the REACH Regulation would discriminate against chemistry as a neutral scientific discipline.

### Consumers’ knowledge and expectations of the ‘SVHC right to know’

Participants were asked in Question 17 whether they knew that they had the right to ask for information about substances of very high concern in articles according to the REACH Regulation (‘SVHC right to know’). Only one out of seven participants (14.7%) stated to be well informed about the ‘SVHC right to know’. Another 30.2% had heard it before, while more than half of the respondents (55.1%) had never heard about it. More than a third (37.1%) of people who are working with chemicals or REACH at their workplace stated to be well informed compared to 3% of persons without this working experience (*χ*^2^ = 299.3, *p* < 0.0001). Nearly three quarters (72.1%) of people who are not working with chemicals or REACH at their workplace have never heard about the ‘SVHC right to know’. Nearly all participants (92.6%) who indicated to be well informed about the ‘SVHC right to know’ have good or very good chemical knowledge (*χ*^2^ = 233.5, *p* < 0.0001). It is interesting that only half of the respondents (50.0%) who had indicated to know the REACH Regulation well (Question 15) stated to know also the ‘SVHC right to know’(Question 17). Even one out of ten (10.0%) of these persons had never heard about the ‘SVHC right to know’. Two-fifths of the respondents (41.6%) had neither heard about the REACH regulation nor about the ‘SVHC right to know’.

**Fig. 2:**
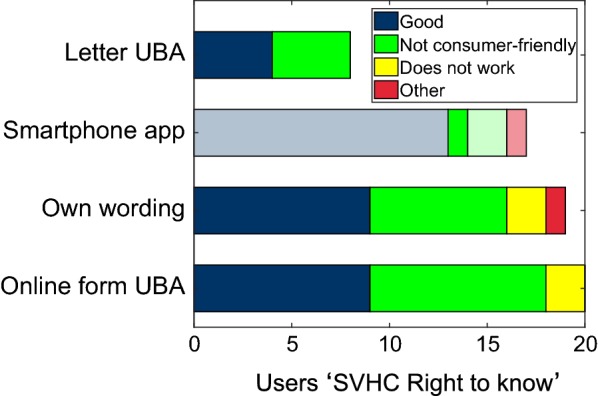
Methods indicated by study participants for the ‘SVHC right to know’ inquiries (Question 18) (multiple replies were possible.). The subjective assessments about ‘SVHC right to know’ given by these consumers in Question 20 are also shown. All but one person who indicated to use smartphone applications did apparently not inquire for SVHCs as there was no smartphone application available at the time when they answered the survey. Therefore, these respondents cannot be considered and the column showing these users of smartphone applications is shaded except one user who replied to the questionnaire after the update of the ToxFox smartphone application and who found the ‘SVHC right to know’ not consumer-friendly

Respondents who had indicated in Question 17 to know the ‘SVHC right to know’ or to have heard about it (together 469 persons) were asked in Question 18 whether they were using it and which methods they would apply. The vast majority (88.6%) of this subgroup indicated not to use the ‘SVHC right to know’. This percentage was lower (60.0%) for members of consumer organizations (*χ*^2^ = 16.9, *p* < 0.0001). Only 55 persons indicated to make inquiries according to the ‘SVHC right to know’. They selected various methods [20 persons indicated to use the online form of the German Environment Agency (UBA), 18 own wording, 17 inquiries per smartphone applications (e.g., ToxFox, see Box 1), and 8 persons indicated to use the sample letter to be downloaded on the homepage of the UBA] (Fig. 2).

Box 1 illustrates various online and smartphone methods available for inquiries about ingredients in consumer products.

More persons made the effort to formulate the inquiry themselves compared to the participants who used a smartphone application or the UBA sample letter. In our survey, 15 persons declared to use a smartphone application for the SVHC requests and had answered the questionnaire before the 20th of October, but, at that time, no smartphone application for the SVHC requests existed in German language. The upgrade of the ToxFox for SVHC requests had just become available on 20th of October, 2016. The smartphone application Scan4Chem was not usable yet for the public, and CodeCheck does not inform about SVHCs. We assume that these 15 study participants did not apply the Danish tool Tjek Kemien [11], as this smartphone application is available only in Danish language. These participants apparently assumed erroneously to have received SVHC data by the basic ToxFox application (which dealt with endocrine disrupting substances in cosmetics only before the upgrade was installed) or by the CodeCheck application. Three of these persons had also indicated to use other methods. Interestingly, 10 of these 15 persons who indicated erroneously to have used a smartphone application for SVHC requests found this method good (Question 20). Therefore, we cannot consider those survey participants, who indicated to use smartphone application as only method, as participants who make SVHC requests. This means that only 42 persons declared to make SVHC requests, which are only 4% of the total of the 1030 participants who had answered the complete survey. We assume that the other methods for SVHC requests indicated by these 42 users were applied correctly. Three persons indicated to use two different methods and one person indicated three different methods (if the users of smartphone applications before the 20th October are not counted).

Three quarters of the users of the smartphone application (76.5%) were between 20 and 39. There were no other significant correlations between the methods used and the personal characteristics. Even persons who were working with chemicals or REACH at their workplace or people with a university degree did not indicate to use the ‘SVHC right to know’ significantly more often that the other groups. Although more persons with good or very good chemical knowledge stated to know the ‘SVHC right to know’ compared to the persons with no knowledge (answers to Question 17), these participants did not use the ‘SVHC right to know’ more frequently than the other participants.

Participants who indicated in Question 17 that they did not know the ‘SVHC right to know’ (597 persons) were forwarded to an information box giving the basic background about the ‘SVHC right to know’ (see Questionnaire in Appendix and Additional file 2: Figure S2). These participants together with the participants who had indicated in Question 18 not to use the ‘SVHC right to know’ (413 persons) were asked in Question 19 whether they intended to use the right to know in future. Only one out of ten respondents (11.6%) affirmed this, women more frequently (12.9%) than men (7.2%) (*χ*^2^ = 8.1, *p* < 0.005). Two-thirds of these persons (66.8%) answered with ‘perhaps’. Some respondents (13.7%) stated that they would not use the ‘SVHC right to know’ in future, because they found it too complicated. Persons who are working with chemicals or REACH at their workplace (7.6%) chose this option less compared to persons without this working experience (13.4%) (*χ*^2^ = 7.1, *p* < 0.008). Another 7.6% did not intend to use the ‘SVHC right to know’ in future, because they would not have the time. There was a clear correlation with age, the younger the participants, the less they would use the right because of lack of time (9.1% in the age group 20–29, to 0.0% in the age group > 70) (*χ*^2^ = 23.2, *p* < 0.0008). In addition, parents with children below the age of 18 indicated the option, that they would not make SVHC inquires because of lack of time more often (9.2%) compared to persons without young children (5.2%) (*χ*^2^ = 5.5, *p* < 0.02). Only 3.5% of the participants have no interest at all to use the ‘SVHC right to know’; interestingly, more persons with very good knowledge in chemistry (7.0%) than persons with less knowledge (2.1 and 2.3%) (*χ*^2^ = 9.8, *p* < 0.007) had no interest.

**Fig. 3:**
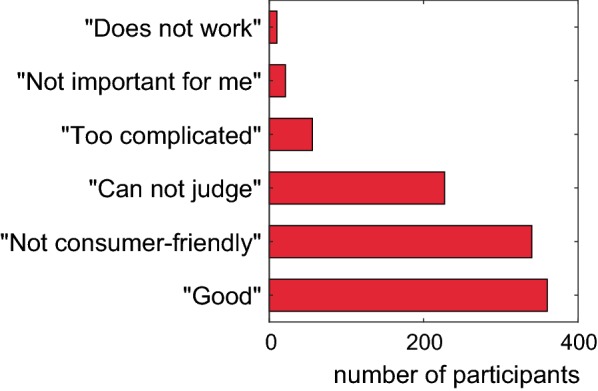
Subjective assessments of the ‘SVHC right to know’ (Multiple replies were possible.). (Question 20)

All participants (independent of their answers in Questions 17–19) were asked in Question 20 to give their opinion about the ‘SVHC right to know’ (Fig. 3).

More than a third (34.9%) chose the option ‘I think it is good’. Some of these (13 persons) added qualifying critical comments which adjusted their positive statements (such as ‘it takes too long before an answer arrives’, ‘one needs a lot of knowledge to understand the answer given by a supplier’, and ‘it would be preferable to have a complete declaration of the ingredients’).

A third of the participants (32.9%) were of the opinion that the ‘SVHC right to know’ is not consumer-friendly and too time-consuming for the everyday routine. The younger the respondents, the more often this answer was given (38.2% in the age group 20–29 up to 18.5% in the age group > 70) (*χ*^2^ = 16.4, *p* < 0.02). Again, some (23) persons who gave this answer made also critical comments (e.g., ‘difficulty for most consumers to evaluate the answers by the suppliers realistically’, ‘need for an extensive information for consumers and distribution of knowledge by media, authorities and associations’, ‘obligation to inform about SVHCs on the packaging instead of right for inquiries’, ‘the threshold of 0.1% is too high’, ‘two worlds collide: legal requirements and moral indignation. A difficult problem of affluence’, ‘distrust towards the suppliers’ answers’, and ‘need of independent controls’).

Another 5.4% found the ‘SVHC right to know’ too complicated. Participants with no self-reported chemical knowledge chose this option more often (8.1%) compared to participants with good (3.4%) or very good knowledge (4.8%) (*χ*^2^ = 8.6, *p* < 0.02). Five critical comments were given in addition to this answer, e.g., ‘very big effort for the manufacturers’.

There were 2.0% of the participants who specified that the ‘SVHC right to know’ was not important for them. Four persons who did not find the ‘SVHC right to know’ important gave additional comments, e.g., ‘refusal of the ‘right to know’ because of animal tests’; ‘missing controls whether the substitutes are less problematic’.

The option ‘the ‘SVHC right to know’ does not work, because companies do not answer’, was selected by 1.0% (10 persons). Among these participants were six persons who had not indicated in Question 18 that they were using the ‘SVHC right to know’. It must be assumed that they based their opinions on the experiences made by other consumers who had inquired for SVHCs. Participants who are working with chemicals or REACH at their workplace indicated more often that the ‘SVHC right to know’ would not work (2.0%) compared to persons without this working experience (0.5%) (*χ*^2^ = 5.7, *p* < 0.02).

Nearly a quarter of the participants (22.0%) declared that they would not be able to form an opinion. This was the case for more participants without knowledge in chemistry (22.7%) compared to participants with very good knowledge (16.3%) (*χ*^2^ = 6.4, *p* < 0.04). Seven respondents made additional, more or less detailed comments. One person understood the principle, but had problems believing it: ‘I do not understand whether this makes sense in the daily routine. Should I ask the supplier for any article that might contain problematic substances and wait up to 45 days for an answer?’

Interestingly, nobody of the persons who had answered in Question 16 to use a smartphone application for their SVHC request chose the option ‘does not work’, although all but one had presumably used smartphone applications which could not answer SVHC requests (Fig. 2).

In Question 21, survey participants could describe their judgement about the overall outcome of the ‘SVHC right to know’. They could choose multiple replies offered and they could add free text. On average, nearly every participant chose one option which considered the ‘SVHC right to know’ negative (31.2% ‘no improved consumer and environmental protection, because it is hardly used’, 23.5% ‘great administrative burden for companies to answer the consumers’ inquiries correctly’, 18.9% ‘great burden for authorities to control’, 7.8% ‘increased feeling of unease in consumers’, 4.6% ‘no improved consumer and environmental protection, even if it is used on a large scale’, and 3.7% ‘nothing’). More participants with very good knowledge in chemistry (36.9%) considered the ‘SVHC right to know’ to be a great administrative burden for companies compared to 19.7% of persons with no knowledge in chemistry (*χ*^2^ = 27.7, *p* < 0.0001). More younger participants declared that the ‘SVHC right to know’ would be a great administrative burden for companies compared to older participants (30.0% in the age group 20–29 down to 14.8% in the age group above 70) (*χ*^2^ = 12.7, *p* < 0.05). The great burden for authorities was seen as an outcome of the ‘SVHC right to know’ by 27.1% of participants with very good knowledge in chemistry compared to 16.4% of participants with no knowledge in chemistry (*χ*^2^ = 12.4, *p* < 0.003).

More men (10.6%) compared to women (6.0%) ticked the option that the ‘SVHC right to know’ would increase the feeling of unease in consumers (*χ*^2^ = 8.0, *p* < 0.005). More men (6.3%) compared to women (2.0%) were of the opinion that the ‘SVHC right to know’ would not lead to anything (*χ*^2^ = 13.8, *p* < 0.0003).

At the same time, nearly all participants chose on average also a positive answer (33.9% ‘improved consumer protection’, 28.8% ‘improved protection of the environment’, and 24.1% ‘reduced use of substances of very high concern by manufacturers’).

Older participants were more optimistic concerning potential positive effects of the ‘SVHC right to know’ than younger persons. Every second person above 70 (57.1%) chose ‘improved consumer protection’, while only a third (29.5%) of the 20–29-year-old participants selected this (*χ*^2^ = 21.9, *p* < 0.002). More than a third of persons above 50 (between 36.2% up to 44.4%) selected ‘improved protection of the environment’ compared to less than a quarter of younger persons below the age of 49 (between 20.9 and 24.1%) (*χ*^2^ = 43.4, *p* < 0.0001) who selected this option.

While 35.1% of the survey participants with no chemical knowledge considered the ‘SVHC right to know’ as improvement for the protection of the environment, only 22.4% of the participants with very good knowledge in chemistry were of this opinion (*χ*^2^ = 13.0, *p* < 0.002).

A quarter of the participants (25.7%) indicated that they were not able to tell what outcome the ‘SVHC right to know’ would have. This was the case for twice as many participants without knowledge in chemistry (29.1%) compared to persons with very good knowledge in chemistry (15.9%) (*χ*^2^ = 13.5, *p* < 0.002).

Comments were given by 42 participants, some of which were again very detailed and showed the competence of these persons. A third of these participants expressed their view, that the ‘SVHC right to know’ would need to be communicated better to be effective. Another third described negative aspects concerning consumer protection (such as ‘the ‘SVHC right to know’ is far from practical reality of a consumer who wants to buy a product when he needs it and does not want to wait for 45 days’, ‘missing controls’, ‘missing sanctions’, and ‘‘SVHC right to know’ is not designed for the consumer, but for professionals’). Nine comments praised the positive features of ‘SVHC right to know’, such as ‘more transparency’, ‘information yield’, ‘increase of awareness’, or ‘increased motivation to avoid SVHCs’. Three participants proposed different solutions, such as the full declaration of the ingredients on the packaging of articles. Two comments referred to the fact that avoidance of certain substances could make products more expensive or lead to disappearance of certain products in the market.

### Box 1. Online information and smartphone applications for inquiries about ingredients in consumer products

The smartphone application ToxFox released by BUND (Friends of the Earth Germany) has been available since 2012 as a tool to inform consumers about endocrine disrupting ingredients in personal care products [12, 13]. The ToxFox smartphone application was upgraded on 20th October 2016, just at the end of our survey, and includes, since then, also SVHC inquiries. The answers received upon each costumer request for a specific article with the ToxFox application are saved and build a growing data base. This allows that a customer will receive the SVHC information stored in the database for an article immediately at the point of sale without having to wait for 45 days. This big advantage of the database goes along with the disadvantage that the database has to be updated regularly, because of the updates of the candidate list and potential changes of article compositions. The ToxFox is being scientifically monitored in the project KinChem until the end of 2018 [14, 15]. At present (28th March 2018), there are 1.35 million downloads of ToxFox via Google Play and iOS (personal communication by Kallee, Friends of the Earth).

The smartphone application Scan4Chem [16] for SVHC requests launched by the German Environment Agency (UBA) on February 17th, 2017, simplifies the inquiry process for consumers by automatically generating and sending requests. At present (14th March 2018), the number of Scan4Chem downloads in Germany amounts to 2900 (personal communication by Becker, UBA).

The Danish smartphone application Tjek Kemien [11] was launched in 2014 by the Danish EPA and the Danish Consumer Council, and was available in Danish language with Danish and English explanations on the homepage. A database is connected to the application which allows companies to save their SVHC data and consumers to retrieve these data.

The EU LIFE project AskREACH (GIE/DE/000738) started on 1st September 2017 and will develop a European smartphone application for SVHC requests coupled with a European data base into which suppliers can enter their SVHC data [17]. This European smartphone application will be available in most, if not all EU Member States and will allow consumers to retrieve SVHC data in the database and to send SVHC requests to suppliers who did not yet make their data available in the data base. The project partners will conduct awareness raising campaigns for suppliers and consumers within at least 18 EU member states during several years.

The smartphone application and the homepage CodeCheck [18] offer information about a multitude of various products based on data entered by consumers. It does not inform about SVHCs. At present (18th February 2018), the number of downloads of CodeCheck amounts to one million according to App Store.

The Danish App Kemiluppen allows consumers to easily recognize the 26 fragrance allergens, and endocrine disruptors in ingredient lists of cosmetics [19].

### Potential acceptance of upcoming smartphone applications

Smartphone applications are expected to have a great potential to improve the effectiveness of the ‘SVHC right to know’ (Box 1). Questions 22–24 were designed to learn about potential users among interested motivated consumers.

The vast majority (81.6%) indicated to have a smartphone. The percentage depended on the age groups (90.4% for the 20–29 up to 55.6% for the over 70 years old) (*χ*^2^ = 62.5, *p* < 0.0001). Very few persons had installed the smartphone applications which give or assist to give information about ingredients in products (see Box 1) (3.7% had installed ToxFox as well as CodeCheck, 5.9% only ToxFox, and 5.0% only CodeCheck [10]). Significantly more women (5.2%) had installed CodeCheck compared to men (2.2%) (*χ*^2^ = 5.2, *p* < 0.03). Most of these survey participants indicated to use these smartphone applications for products which they intended to buy (83.5%) or for products which they have already bought (67.8%), while also a minor portion indicated to use it for products which they do not plan to buy (7.4%). There were also participants who did not use the smartphone applications which they had installed (8.3%).

### Consumers’ handling of SVHC-containing articles

In Question 25, participants could indicate whether they would buy an article with SVHCs. Only 1.0% (among them was no member of an environmental organization) stated that they would purchase the product nevertheless. Half of the participants (53.6%) indicated that they would not buy an article, if they were informed by the supplier that it contained SVHCs, while the other half (45.4%) indicated that it would depend on the articles, what they would do, if an article contained SVHCs.

Two-thirds of the persons (66.7%) (*χ*^2^ = 66.6, *p* < 0.0001) who had no self-reported chemical knowledge stated, that they would not buy the articles, and one-third (31.6%) declared, that it would depend on the article. It was the other way round for persons with very good self-reported knowledge in chemistry: One-third of them (33.5%) stated that they would not buy the articles, while nearly two-thirds indicated that it would depend on the article (61.7%).

**Fig. 4:**
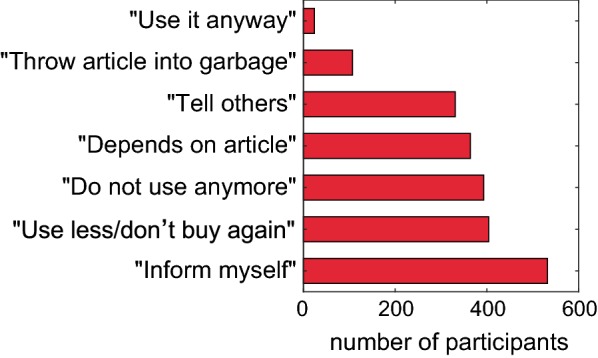
Reactions by participants to SVHC information. Participants could indicate what they would do if they learned about the presence of SVHCs in an article that they have already purchased (Question 26) (Multiple replies were possible.)

In Question 26, participants could indicate, what they would do with an article they had already purchased, if they learned that it contained SVHCs (Fig. 4).

Every second participant (52.2%) stated that he or she would gather more information about the respective SVHC. Young people were more interested in searching information about the SVHC than older ones (58.6% in the age group 20–29 down to 38.9% for persons above 70, *χ*^2^ = 36.2, *p* < 0.0001). Students and persons in job training indicated to have the highest interest (72.0%) out of all professional groups (*χ*^2^ = 26.8, *p* < 0.0001).

Two-fifths (39.6%) declared that they would use the article less frequently and not purchase it the next time. Again young people declared this most often (50.5% in the age group 20–29), compared to older people (e.g. 27.8% for over 70, *χ*^2^ = 34.5, *p* < 0.0001). About the same number of persons (38.6%) stated that they would no longer use the article. This answer was given preferentially by people with no chemical knowledge (47.0%) compared to people with good (37.0%) and very good knowledge (23.8%) (*χ*^2^ = 31.5, *p* < 0.0001). Young people indicated this less frequently (20–29: 28.2%), compared to older participants (maximum 50–59: 44.8%) (*χ*^2^ = 15.8, *p* < 0.02). Around a third (35.7%) stated that it depended on the article, what they would do. This option was again preferred by persons with very good knowledge in chemistry (46.3%) compared to persons with no self-reported knowledge (27.4%) (*χ*^2^ = 24.3, *p* < 0.0001). A third of the participants (32.5%) declared that they would inform other people, women to a larger percentage (36.9%) compared to men (25.1%) (*χ*^2^ = 13.6, *p* < 0.0003). In addition, more participants with no knowledge in chemistry (36.3%) (*χ*^2^ = 6.5, *p* < 0.04) and more young persons compared to the other demographic groups stated that they would inform other people (20–29: 44.5%) (*χ*^2^ = 20.6, *p* < 0.023). One out of ten (10.5%) chose the option that he or she would throw the article into the garbage. Members of environmental organizations declared this more frequently (14.0%) (*χ*^2^ = 5.1, *p* < 0.03). A small number of participants (2.4%) indicated to use the article, nevertheless.

Additional suggestions were made by 51 (5.0%) participants. Members of environmental organizations (9.3%) (*χ*^2^ = 14.1, *p* < 0.0002), members of consumer organizations (13.5%) (*χ*^2^ = 5.8, *p* < 0.02), and persons with chemical intolerance (9.0%) (*χ*^2^ = 11.5, *p* < 0.0007) were more prone to add their own options than the other demographic groups. Most of the persons who made comments (22 persons) wrote that they would dispose of this article in accordance with the regulations; among them were seven who wrote that they would dispose of these articles in the hazardous waste collection sites. Nearly as many persons (17) would return the article to the manufacturer. Two would sell it to someone else. Further comments showed differential views of some participants: ‘one must assume that all articles contain SVHCs.’ ‘It depends on the exposure.’ ‘It depends on whether there are alternatives.’ ‘One has to weigh the pros and cons.’ ‘It depends on the specific SVHC’.

### Improvements suggested by study participants

**Fig. 5:**
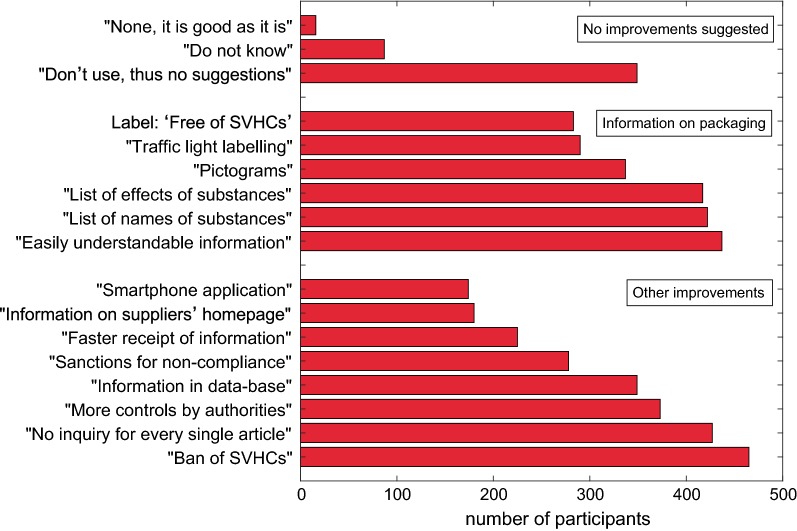
Improvements suggested by the participants concerning the ‘SVHC right to know’ (Multiple replies were possible.) (Question 27)

In Question 27, participants could choose out of 18 suggestions for improvement of the ‘SVHC right to know’ (Fig. 5). Altogether, 5131 responses were given.

Only 1.6% of the study participants declared that the ‘SVHC right to know’ is good as it is. A third of the participants (33.9%) indicated that they did not use it and had, therefore, no suggestions for improvements, and 8.4% of the participants did not know, what to answer. All the other participants suggested improvements or alternatives.

Nearly half of the respondents (45.1%) wished that substances of very high concern should be prohibited as soon as possible. This was suggested more frequently by persons without self-declared knowledge in chemistry (49.3%) compared to persons with very good knowledge (35.0%), (*χ*^2^ = 10.8, *p* < 0.005). Young persons in the age class 20–29 chose this option less (31.8%) than persons in other age classes (up to 52.5%) (*χ*^2^ = 20.8, *p* < 0.002). Most suggestions for improvements concerned a better information on the packaging. On average, every participant gave two suggestions to improve the information on the packaging. The favourite proposal given by 42.4% participants asked for easy to understand information about all harmful substances on the packaging. Two out of five (41.0%) participants wished to have the SVHC names on the container. In addition, two out of five participants (40.5%) wished to see the effects of the SVHCs on the packaging (more persons with chemical intolerance, 52.9%) (*χ*^2^ = 4.6, *p* < 0.04). A third of the participants (32.7%) desired a pictogram, which indicates the presence of an SVHC. A traffic light labelling system (green meaning ‘SVHC < 0.01%’, yellow meaning ‘small concentration of one SVHC’, and red meaning ‘larger amount of several SVHCs’) was desired by nearly a third of the participants (28.2%), and slightly less (27.5%) wanted to have a notice: ‘This product is free of substances of very high concern.’ This option was preferred by more women (31.9%) than men (21.0%) (*χ*^2^ = 12.7, *p* < 0.0004).

A third of all answers concerned various improvements of the ‘SVHC right to know’ other than information on the packaging. Two out of five participants (41.5%) did not want to make an inquiry for every single article. One out of five participants (21.8%) wanted to receive the answer to the SVHC request faster and not after a period of 45 days maximum. Only one out of six respondents (16.9%) desired a smartphone application which shows directly in the shop on the basis of the barcode, whether the article contains substances of very high concern and which effects these might have. There was no correlation with age, although more young people have smartphones than older ones (Question 22). Slightly more participants (17.5%) wanted to have information about SVHCs on the homepage of the manufacturer.

More than a third of the participants asked for better controls by authorities (36.2%). Less than a third (27.0%) wanted that fines were applied more often to companies in case they do not meet their information duties. However, one person added that improved protection should not lead to more bureaucracy.

A third of the participants (33.9%) wanted to have easy to understand information about all harmful substances in publicly available databases.

Again, the open answers to Question 27 given by 22 participants showed the high level of knowledge of these persons. Six participants made detailed suggestions for a better declaration of ingredients in consumer products (e.g., ‘harmonization of labelling’, ‘no exceptions for special product groups’, and ‘full declaration of ingredients’). Five comments asked for a complete ban or a minimization of SVHCs. Three comments requested more controls, e.g., ‘by independent institutions’, and ‘effective sanctions for suppliers in case of non-compliance’. Three participants addressed the difficult definition of SVHCs and wrote that more substances should be added to the list, or that it should be described in simple words what SVHCs are. More information for the public about the REACH Regulation and the ‘SVHC right to know’ was demanded by another three comments. Further comments asked for a more differentiated approach with chemicals or problematized that a general ban of SVHCs could cause new problems with substitutes and lead to increased costs.

The survey participants could add general comments in the final Question 38. Every second of the 66 comments added was very positive, thanked for the good survey and the information received or wished us good success. The great interest in this study was also reflected in the large number of persons (a third of all participants) who sent us an e-mail, indicating that they would like to be informed about the final results of the survey and to take part in the lottery (see Questionnaire in Appendix). There were 20 comments concerning the design of the survey, which were very heterogeneous, some found it too detailed, others too short, some wrote that they had technical problems adding comments, while others added comments up to 3600 characters, some considered the survey as very differentiated, while someone else looked at it as small talk, some found the questions tendentious, others found them very good and easy to answer, someone would have liked to give more detailed information about his or her education level, and another participant missed a third sex besides women and man in the demographic section. Some comments addressed new ideas: someone described his or her impression that the critical examination of environmental issues is misused for cheap showmanship in many cases. Another participant asked us to stay objective in the analysis of the answers.

## Discussion

### Very few participants use the ‘SVHC right to know’

**Fig. 6:**
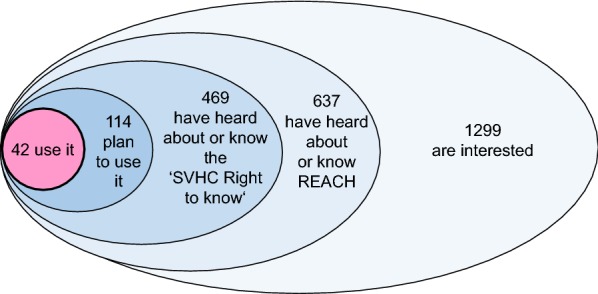
Visualization of the subgroups (not to scale). In Question 1, 1299 participants declared that they were interested in harmful substances in consumer products; in Question 15, 637 indicated to know the REACH Regulation well or to have heard about it; in Question 17, 469 of this subgroup indicated to know the ‘SVHC Right to know’ well or to have heard about it; in Question 19, 114 declared that they intended to use it in future; in Question 18, 42 were actually using it

A high number of SVHC requests made by consumers could be a relevant incentive for suppliers to substitute SVHCs and would hence increase the effectiveness of the ‘SVHC right to know’ on the way to a non-toxic environment. The refusal by half of the participants to purchase SVHC-containing articles (Question 25) could really be a strong stimulus for suppliers to substitute SVHCs by substances of less concern or by non-chemical alternatives. However, as long as only 4% of the highly motivated and interested participants make SVHC inquiries (Question 18, 19) (Fig. 6), the number in the average population will be even lower and the stimulus for substitution caused by the SVHC inquiries remains very low at present.

Nearly all participants are interested in general (Question 1); half of them stated that they would like to learn more about the SVHCs in their articles at home (Question 26), but only one out of ten intended to make the SVHC inquiry (Question 19). This means that a general interest in harmful ingredients does not necessarily go along with an inquiry for SVHCs in a concrete article. This coincides with the results found in a study conducted on the basis of 172 oral interviews and 256 questionnaires about consumers’ awareness of and opinion about chemical ingredients in textiles in 2008 [20], where the majority of the respondents (70%) said that they would like to know more about the ingredients in textiles, but only 30% said that they would inform themselves before they purchase a product. The high interest of consumers in ingredients of articles was also shown in the results of a comprehensive survey in all European member states, where 67% of the respondents in Germany had indicated to check the ingredients before purchasing in toys, 51% in clothes, 38% in furniture, and 25% in electronics [21]. These results support our finding that consumers would like to know more, but tend to avoid the burden of making investigations.

### The job and chemical knowledge make a difference

Four out of ten consumers who indicated to be interested in harmful substances in their everyday consumer products had never heard about the REACH Regulation, and five out of ten had never heard about the ‘SVHC right to know’. This implies that this communication instrument reaches only a minority of consumers, not even all interested ones.

In contrast, most members in the subgroup of participants who are working with chemicals or REACH at their workplace knew the REACH Regulation well or had heard about it, and every second of this subgroup considered the REACH Regulation as ‘a great step forward’. More than every third participant working with chemicals or REACH at the workplace knew the ‘SVHC right to know’ well, whereas this was the case for only 3% of participants without such a job.

In our previous study, we had found out that knowledge in chemistry helped, but was not enough. There were also participants with good knowledge in chemistry who made wrong assumptions, e.g., some of them assumed that products with an eco-label, natural personal care products, products without hazard pictograms, or products produced in the European Union would not contain harmful substances [10].

People with good chemical knowledge understood better that SVHC-containing articles do not necessarily pose an immediate risk and do not need be disposed of straight away. More of them would still use the article or decide depending on the specific article what they would do. Interestingly, people with good chemical knowledge tended less to inform other people about articles that contain SVHCs than persons without knowledge in chemistry (Question 26). They understood the matter better than other demographic groups, but apparently did not find it necessary to circulate this information or they are reluctant to try to communicate this complex matter.

### The ‘SVHC right to know’ is for everybody

The answers to all but one question depended on demographic factors, such as on chemical knowledge, education, age, or other parameters as described in the “Results” section. This coincided with our previous results [10], where we had found substantial differences between the answers given by various demographic groups and had concluded that differences between individuals must be taken seriously.

Interestingly, there was one single case where no correlation with personal characteristic could be detected. Our hypothesis had been that people who know much about chemistry or work with chemicals or REACH would use the ‘SVHC right to know’ more frequently, because nearly all participants who indicated to be well informed about the ‘SVHC right to know’ have good or very good chemical knowledge. However, there was no correlation between chemical knowledge or experience with chemicals and usage of the ‘SVHC right to know’. Apparently, people with good knowledge in chemistry have other means to inform themselves, so that this information instrument does not play a big role for their personal risk assessment of everyday articles. It is also possible that these persons purchase preferentially green products, which contain less harmful substances or minimize their overall consumption.

We would also have expected more and stronger correlations with demographic factors in the proposals for improvements (Question 27).

These missing correlations are in agreement with the results obtained in the survey of consumer information about textile ingredients, where the information strategies and assessments of chemicals by the respondents were independent of the demographic variables like gender, age, educational level, or income [20]. While Steffensen [20] concluded that it would not be necessary to establish various specific sources of information for various groups in the population, our results would not, in general, support this, because there were clear differences of the various demographic groups in most answers. This outcome shows that more studies of this kind are needed to be able to refine the needs for optimal information strategies.

### The REACH Regulation and the ‘SVHC right to know’ are complicated

It is interesting that only half of the respondents, who had indicated to know the REACH Regulation well, stated to know also the ‘SVHC right to know’. Every tenth of these persons had even never heard about the ‘SVHC right to know’. The REACH Regulation is a very complex regulation with various detailed provisions, so that, in fact, persons who seem to know the Regulation well are not familiar with all details.

The complicated nature of the ‘SVHC right to know’ seems to be a conscious obstacle only for a minority (Question 16 and 21), but it is not certain whether all other participants had understood its details and its complexity fully. This became evident by the survey participants who had indicated to know the ‘SVHC right to know’ well and would use a smartphone application which—at that time—was not designed to make SVHCs requests (Question 18). Another example are the experiences obtained with SVHC requests via ToxFox, which showed that half of the inquiries by consumers were made for food products, which are exempt from the ‘SVHC right to know’, as well as cosmetics and other mixtures [22]. It must be questioned whether these consumers knew that they could make the SVHC inquiry for the packaging of food or cosmetic products, because the containers are articles as defined in the REACH Regulation and suppliers of food or cosmetic products should inform consumers about the SVHC content in the containers upon an SVHC request. It is also not clear, how many consumers know that the presence of SVHCs does not mean, that there are immediate risks for their personal health associated with the use of the respective articles. The consumer is left alone with the interpretation of this information, as shown in the answers to Question 26. In fact, the right to know is rather a ‘hazard communication instrument’ which informs about a possible source of danger and not a ‘risk communication instrument’, because the consumer does not receive information about the exposure. A reasonable risk assessment would require details about the potential effects together with the exposure [release rate and concentration of the substance in the article (which can be just above 0.1% or up to 100%), use frequency of the article, aggregated exposure from various sources, and so on] or discharge into the environment, and could only be assessed by an expert who invests time and efforts. In the present survey, only one respondent mentioned the aspect of exposure (Question 26). In addition, according to the precautionary principle [1], the discharge of all substances which are very critical for the environment, because they are persistent, bioaccumulative and toxic or very persistent and very bioaccumulative should be minimized. It is also not clear, how many survey participants understood, that suppliers do not need to reply to an SVHC request, in case the article does not contain any SVHCs above the threshold. If no SVHCs are declared, they could still be present in the article below the threshold. One participant wrote (Question 26) that nearly all articles contain SVHCs, which is probably true for most electric and electronic articles, motor driven vehicles, or furniture, and illustrates, why the reaction to discharge any SVHC-containing article with the hazardous waste as suggested by several participants (Question 26) would be a disproportionate reaction. Many consumers seem to think that an article that contains SVHCs is very dangerous and needs to be discharged (Question 25 and 26), not realizing that the risks for their health and for the environment depend not only on the SVHCs, but also on the properties and amounts of the additional other harmful substances present in an article or product (as explained in [10]).

Further complications result from the fact that the candidate list is still growing ([4], see also SVHC Roadmap [23]), which makes it difficult for manufacturers to comply with the provisions and for authorities to enforce them [8]. There are various parties who suggest that the candidate list should already be much longer, e.g., like the compilation by Kalberlah in 2011 [24] or the SIN list developed by ChemSec (‘substitute it now’ [25]) which has identified 913 potential candidate substances so far. Another complex issue is the selection of suitable substitutes for SVHCs in an article that are clearly of less concern. The SINimilarity tool developed also by ChemSec [25] offers help to avoid substitution by substances of similar hazard. Another aspect which complicates the situation is that the SVHC communication provisions should also be applied to imported articles, but the enforcement is even more difficult than for articles produced in the EU [26]. Therefore, the comment by one survey participant who asked for a more differentiated approach  for chemicals in articles is justified, but it shows again the complicated nature of the topic.

The fact that many participants did not feel able to form an opinion, even after the ‘SVHC right to know’ was explained to them in this questionnaire, shows how difficult it is even for motivated consumers to decide (Question 20 and 21) and it explains why two-thirds were not sure, whether they would use the ‘SVHC right to know’ in future (Question 19). One person explained why he or she found it difficult for consumers to give their opinion, as long as chemicals are demonized and the information for consumer is insufficient. He or she added that consumers should be able to trust, but, in fact, they cannot. Another participant described that—according to his or her experience—products, which are declared to be environmental friendly perform less compared to the conventional products and can lead to even an increased use of resources in the end.

It is also alarming that apparently many survey participants were not aware of the complexity of the ‘SVHC right to know’, while, at the same time, three quarters were of the opinion that consumers carry the responsibility for the reduction of substances harmful for human health and the environment [10]. One participant (Question 38) even wrote that consumers should not be patronized, he or she wrote, that consumers should always be able to make their own free decisions on the basis of information received by the authorities according to the slogan ‘rat poison for everybody’. However, many answers in this questionnaire (e.g., Question 26) reveal that too much is expected from average consumers, and that most of them are not capable to assess the effects of SVHCs and other harmful substances in their articles without help.

It must be noted that the ‘SVHC right to know’ communication tool is special, as it does not comply with the classic straightforward ‘knowledge deficit model’ [27], which assumes that sufficient information will automatically lead to the desired behavior of the recipient of the information. In the case of the ‘SVHC right to know’, the desired behavior is primarily not on the side of the information recipients, who are the consumers, but, on the side of the providers of the information, the manufacturers, who should be motivated to substitute or eliminate the SVHC. The potential reactions of study participants when they were informed about SVHC presence (Question 25 and 26) also show how difficult it is to transfer sufficient information to consumers for suitable risk behavior. Even the desire by some participants to receive an easy to understand definition of SVHCs is not easy to fulfill.

### Interest in smartphone applications is low

There are huge efforts to improve the SVHC requests by smartphone applications (Box 1), but the participants in this survey had only minor interest in these applications (Question 8, 23, 24, 27). The rare use of smartphone applications was not expected in this group of highly motivated participants. However, it can be assumed that the number of the persons who use ToxFox, Scan4Chem, or the future European smartphone application for the SVHC requests will grow with the support given by the KinChem project and the EU Life Project (see Box 1) [14, 15, 18].

This hope is based on the experiences in Denmark, where the request numbers increased after the introduction of the smart phone application Tjek Kemien [11]. Only one out of six respondents (Question 27) desired smartphone applications for their SVHC inquiries; this was the least suggested improvement for the ‘SVHC right to know’. Even slightly more participants wanted to have information about SVHCs on the homepage of the manufacturer (Question 27). Nearly twice as many wanted to have easy to understand information about all harmful substances in publicly available databases. Only less than 10% each stated that they would use the smartphone applications ToxFox or CodeCheck for information about ingredients in everyday products (Question 8), even though the users of these applications considered these information sources as trustworthy (Question 10) [10]. Nearly all persons, who indicated to use a smartphone application for their SVHC requests, had answered the questionnaire at a time when the ToxFox application was not upgraded yet, which means that they could receive only information about ingredients with endocrine effects in cosmetic products by this application and no information about SVHCs in articles. This shows that the correct use of such smartphone applications requires more explanations and support than expected.

A small ray of hope: two participants added that they would download the apps directly after the survey.

The small groups of participants, who make SVHC inquiries, do not clearly prefer one of the methods for the SVHC inquiries (Fig. 2) (Question 18). The online form of the German Environment Agency (UBA), which is no longer active on the homepage of the UBA today, was indicated as method used by around a third of these users. A striking result was that nearly as many users write their own wording and send this inquiry by postal letter or by e-mail to the suppliers. This fact shows again the high commitment and knowledge of the participants of this survey. It was also not expected that the individual wordings were chosen more frequently than the inquiries by smartphone application or by the UBA sample letter.

The ‘SVHC right to know’ informs  only about the presence of SVHCs in an article and does not replace a differentiated assessment of all chemicals in an article. This is probably one of the reasons why one participant declared that the two smartphone applications would increase the feeling of unease in consumers, a feeling that one in ten participants also see as a consequence of the ‘SVHC right to know’ (Question 21). One participant considered the development of a smartphone application by authorities even as a waste of tax money (Question 28).

We had expected that some consumers would address the aspect of personal data protection, as consumer requests by mail or letter involve the disclosure of the consumer’s name and address to the supplier for each single article request. However, nobody mentioned this as a problem. Apparently, there is no relevant awareness of personal data protection in this group of citizens or they are not aware that the SVHC request would demand their names and addresses.

### Young people are interested and concerned, but only a few inquire for SVHCs

The age group between 30 and 39 was the best informed about the REACH Regulation (Question 15), and young people indicated to be more interested in searching information about SVHCs than older age groups (Question 19). Students and persons in job training had the highest interest out of all professional groups (Question 19). This result could be due to the fact that several students who had attended lectures on harmful substances by the author (Klaschka) took part in the survey. Another positive aspect of the participants in the age group 20–29 was the finding that the self-reported knowledge in chemistry was similar for women and men, while, in all other age groups, women indicated to have less knowledge compared to men of the same age class, showing that young women might catch up with the knowledge in this natural science in this specific subpopulation (Additional file 1: Figure S1).

Furthermore, the facts that young people seemed to see less positive effects of the ‘SVHC right to know’ (Question 20), and that they shared information on SVHCs in articles (Question 26) more than older persons, could be attributed to their higher interest and higher concern compared to the other demographic groups in this study. Interestingly, less young people desired the ban of SVHCs (Question 27), which might be explained by the usually good health situation of the younger generation and their difficulty to imagine that this could change.

Our hypothesis was that young people, who have more smartphones compared to older participants (Question 22), would preferentially use the smartphone applications for the SVHC requests (Question 18). Three quarters of the presumed users of the SVHC smartphone application were between 20 and 39, but as described before, they apparently did not realize that they were not asking for SVHCs in articles, but for endocrine disrupting ingredients in cosmetics. This might be an indication that digital natives might be easily familiar with smartphone tools without correctly understanding the information which they receive. Interestingly, young people do not desire to have smartphone applications for the SVHC requests more frequently than older age groups as an improvement of the ‘SVHC right to know’ (Question 27). Apparently, the higher commitment, better knowledge, and special skills with digital media do not go along with the desire to make the effort of SVHC inquiries. In fact, more young people were even of the opinion that the ‘SVHC right to know’ was not consumer-friendly and too time-consuming compared to older age groups. This is another evidence for the difficulty to tell on the basis of questionnaires, whether a declared interest is a strong enough motivation to take action and search for information. With these results, it is doubtful whether the high interest of young participants in combination with their high affinity to smartphone applications (Question 10) will be sufficient incentive for them to use smartphone applications for SVHC requests more frequently in the years to come.

In comparison, in the survey conducted about ingredients in textiles, young persons were described as less interested in information about ingredients and as less concerned [20]. The contrast to our study might be due to the selection of the survey participants. While Steffensen et al. [20] made a random selection in the streets, the present group of survey respondents consisted mainly of interested and motivated persons [10], and the young participants here seemed to be even more motivated and interested than the older age groups.

### Information on SVHCs can be a trade obstacle

Companies that take their responsibility seriously, comply with their information duty and eliminate SVHCs in all their articles are better prepared for upcoming restrictions and authorisation requirements can build trust in consumers and thus can profit from the resulting competitive advantage. However, the REACH regulatory fitness and performance programme evaluation (REACH REFIT) revealed that some retailers perceive their efforts to be well prepared for SVHC consumer requests as superfluous, as the interest by consumers for such information remains low [28]. Several results of our survey make it understandable, why many suppliers are reluctant to inform about SVHCs in their articles [5–8]: only a minority of all consumers would buy SVHC-containing articles with no strings attached (Question 25) and there were even consumers who regard the presence of an SVHC in an article as reason to return it to the retailer (Question 26). However, as long as only 4% of the highly motivated and interested participants make the SVHC inquiries (Question 18, 19), the pressure applied by the consumers’ request to suppliers remains probably lower than other stimuli to substitute SVHCs in articles, such as the sun set date and the authorisation requirement according to REACH Art. 56, 58.

Suppliers have to face several problems in relation to the ‘SVHC right to know’ such as the growing candidate list, the problem of complex articles with a large number of subassemblies, or the efforts needed for the maintenance of a data base as foreseen for the ToxFox smartphone application. Some companies may not be aware of SVHCs in their articles, even though there is a duty to communicate SVHCs in the supply chains pursuant to REACH Art. 33(1). According to the newly updated Waste Framework Directive [29], suppliers will be obliged to report SVHC in articles to an ECHA database (Art. 9) which will imply additional work for the companies.

This concern of a great workload is also reflected by the present survey, where nearly a quarter of the survey participants were of the opinion that the ‘SVHC right to know’ would be a great administrative burden for companies, while a fifth was of the opinion that it would be a great burden for controlling authorities (Question 21). However, some participants made explicit comments expressing their views that the enforcement by authorities was not sufficient and that companies that do not comply with the regulations would remain undetected (see also [8]).

### Improvements are needed

The very small minority of interested consumers in this survey who used their market power and make SVHC requests indicate that the ‘SVHC right to know’ is not a very efficient instrument in influencing the market. Only a tiny fraction of the study participants considered the ‘SVHC right to know’ good as it is (Question 27). Survey participants with very good self-reported chemical knowledge had even a more skeptical view than the other demographic groups (Question 21). The European authorities and member states know that improvements are needed in the legislation of chemicals, but, apparently, some of them see the needs in other areas, not in the case of the ‘SVHC right to know‘, as it says in the final report for the strategy for a non-toxic environment of the Seventh Environment Action Programme [2, 3]: The ‘lack of specific information requirements on toxic substances in articles (except on SVHC) is a problem’ [30]. However, the recent comprehensive survey of REACH REFIT [31, 32] identified important gaps in implementation and needs for improvements [28]. Many of the results described there, support answers obtained in our survey, for example the desire for companies that ‘information requirements on SVHC in articles should remain manageable’ or that ‘information on SVHC in articles should be improved and better communicated’ or the different views from NGOs compared to the industry view.

In the present survey, four main reasons became apparent, why most consumers—even the motivated and interested ones—do not use the ‘SVHC right to know’: many of them just do not know it [only one out of seven indicated to be well informed (Question 17)]. Second, most of them do not want to use it [nine out of ten do not plan to use it (Question 19)]. Third, it is too complicated (see “The REACH Regulation and the ‘SVHC right to know’ are complicated” section), and finally, most of the consumers do not trust information given by manufacturers or authorities (Question 10) [10].

On the basis of the results of this survey, we can propose ways to make the ‘SVHC right to know’ tool more attractive to interested consumers:

The first step would be to raise interest and awareness among consumers and to make them understand that chemicals and especially SVHCs can be present in consumer articles. Twenty-two persons indicated in Question 1 in our survey that they were not interested in harmful substances, and five of them stated that they would also never be interested (Question 2) (Additional file 2: Figure S2) [10], while the other participants of this group identified reasons which could raise their interest: ‘in case I or a family member would suffer from chemical intolerance’ (seven persons), ‘if I had less other problems’ (six persons), and ‘if I knew more about chemistry’ (three persons). The other reasons (‘if I would have to know more about it for job reasons’; ‘If my family and my friends would be interested’; ‘If I had more time’; ‘Other reasons’) were selected only by up to three persons. These answers show that it would not be easy to raise interest in consumers who are not intrinsically motivated and interested. A comprehensive survey conducted in 2010 assessed the perception and understanding of chemical substances by citizens in the European member states and had shown that most respondents feel only moderately or not well informed about the risks associated with chemical products [21], while many Europeans are concerned about chemicals in products, as was shown in a survey conducted by ECHA in 2017 with nearly 28.000 participants: 45% totally agreed and 39% tended to agree that they were worried about the impact of chemicals in everyday products on their health. Even slightly more survey participants were worried about the impact of chemicals in everyday products on the environment (49% totally agreed and 41% tended to agree) [33]. Consumers have ‘the right to know’ and ‘the right to comprehend’ [34]. In our survey, many participants desired that the information offered to consumers should be easily understandable (Question 27). The high number of survey participants, who spread the word, if they have an SVHC-containing article (Question 26) and the important role friends and family play as highly trusted risk communicators (Questions 9, 10) [10] support the importance of easily understandable hazard and risk communication. Education campaigns to change this will not be easy and cannot yield measurable improvements quickly, but they would be extremely beneficial in the long term for the health of the consumers, as well as for the protection of the environment and for the economic benefits for healthcare and pollution control. A good start on the way to a better risk communication is the recently released online platform by ECHA about chemicals in our life [35] and the AskREACH project [17]. We recommend to start information campaigns for the general public at the early age, e.g., at school. Information campaigns would also imply that consumers understand that they have a benefit if they know more (see also Box 2 in [10]). More surveys are required that detect which information reaches the consumer and which important elements are ignored or misunderstood to be able to improve the communication and to implement new communication strategies.

In a second step, consumers should be informed and motivated to inquire for SVHCs in articles. One crucial idea of the ‘SVHC right to know’ is that consumers could exert pressure by their market power on the suppliers, who would then be stimulated to gradually substitute SVHCs in their articles. Definitely more SVHC requests would be required to render such an incentive effective. Many survey participants demanded in their answers to several questions that the knowledge about the ‘SVHC right to know’ should be spread in the public. Only 3% have no interest at all in the ‘SVHC right to know’ (Question 19), which shows that the large number of interested consumers could in principle be motivated. The public should be informed about the objective and procedure of the ‘SVHC right to know’ and receive easy access to consumer-friendly instruments which allow them to effectuate the SVHC inquiry straightforward and if possible at the point of sale without delay [14, 15]. In the present survey, the smartphone applications were used only by few respondents for general information requests about products (Questions 10) or for the SVHC inquiry in articles (Question 18). The new ToxFox and Scan4Chem developments, the outcome of the research project KinChem [14, 15], and the European Life project AskREACH [17] might increase the user frequency. The availability of sample letters for the request, such as the form offered by UBA [36], and the UBA online tool which has been deactivated end of 2016, would certainly remain useful for consumers who do not prefer smartphone applications. Every second participant of the survey was interested in knowing more about an SVHC (Question 26). The high commitment of the study participants, the great number of diligently written, differential comments received (nearly all based on facts and explanations), which went along with an alarming feeling of insecurity among many participants, support our conviction, that it is worth to improve the communication with consumers and to give more attention to their interests and abilities. Consumers should not only receive the name of an SVHC upon request. More detailed information in selectable steps with increasing information depth depending on the consumer’s level of interest should be publically available without the need for disclosure of consumers’ personal data. A first start would be the SVHC response sample letter for suppliers as proposed in [8], where the consumer receives information in addition to the name of the SVHC present in an article (such as concentration present, function, reasons why the substance was taken up in the candidate list, authorisation requirement, classification and labelling, potential substitutes and properties, safety instructions for use and disposal, and other substances classified as dangerous according to the European Regulation on Classification and Labelling [37]) present in an article, and other European legal provisions applicable to the article. It would be useful to take up this sample letter in the next revision of the REACH Regulation document, to spread it by the competent authorities or make it available at the REACH helpdesks. This information could be further improved in an online tool by offering various levels: first level: simple pictogram and easily understandable recommendations for a safe use and disposal of such an article to minimize the personal risk and the risk for the environment (see [20]); second level: names of the substances, concentration, and function in the article; third level: simple description of toxic and ecotoxicological effects of the substance; fourth level: detailed description of the effects of the substance, description about the potential exposure, and substitutes with effects and function. This might seem to be an extreme effort, but with the upcoming restrictions and authorisation requirements according to REACH Art. 56, 58 and the efforts for a non-toxic environment, the number of SVHCs in articles in the future should go down considerably making such an information procedure manageable for the remaining SVHCs in articles.

The third step should consist in building trust. The success of any risk information instrument depends to a large extent, on how trustworthy an information source is considered [10, 20]. More than half of the consumers said that they would not trust the information given by the producers in a survey conducted on chemicals in textiles [20]. In our survey with motivated and interested consumers, even less than 20% of the survey participants had confidence in information provided by manufacturers [10] (Question 10). This distrust could be one of the reasons, why consumers are reluctant to make the effort and search for information given by suppliers. Transfer of transparent correct information in a language that is understandable to consumers could help to increase trust. A better information transfer in the supply chain pursuant to Art. 33(1) [1] would make it more plausible for consumers that companies actually have the knowledge and control over SVHC in their articles, and hence could also help to build up trust. More independent controls could motivate companies to improve transparency about the composition of their articles.

A smartphone application or an information platform which include ALL commodities together (not only articles, not only cosmetic products, but all types of consumer products) would be a huge challenge, but would be very useful for consumers who have difficulties to learn the various specific legal provisions for the various product groups and articles.

As long as the acceptance of the ‘SVHC right to know’ is not increasing considerably and as long as SVHCs may still be present in consumer articles, additional approaches should be pursued. In this survey, SVHC information on the packaging was by far the most preferred information source at the point of sale (Questions 27). Especially, ingredient lists enjoyed great acceptance. Three quarters of the study participants indicated to use them as information source (Question 8) and more than half of the study participants trusted them (Question 10) [10]. The full declaration of ingredients of an article on the container or on an instruction leaflet as described previously could be a successful advancement [8, 10, 26]. This was the favourite proposal for improving the ‘SVHC right to know’ given by nearly half of the participants (Question 27) and mentioned by several persons who made comments, while there was no clear favourite among the various other options for information on the packaging (pictograms, names of SVHCs, traffic light system, etc.) (Question 27). Such a full ingredient list should include all ingredients above a concentration of 0.1% (or below if there are lower thresholds in other regulations) and could be displayed on the containers in a similar way as on cosmetic products [38]. This idea of transparency about ingredients in products is nothing new, but corresponds to the initiative of the Chemicals in products programme (SAICM) pursued by the United Nations [39]. However, ingredient lists as exclusive hazard communication instruments are not consumer-friendly as they burden the consumer with the responsibility to understand the names and assess what the presence of a substance means in a certain product, as shown for personal care products [40]. Ingredient lists are also difficult to implement due to knowledge deficits of article suppliers about all the substances in their articles and due to space limitations on the containers, especially for complex products. Therefore, these suggestions should not replace the best strategy of all: the substitution of SVHCs by less harmful alternatives or non-chemical solutions as soon as possible. Nearly half of the respondents wished that SVHCs should be prohibited (Question 27). In 2018, the European Commission has strengthened the importance of substitution of SVHCs in the comprehensive REACH Review (Action 5 [28]). The ban of SVHCs is also in favour for persons who are not interested in the ingredients in their consumer products, but, of course, have also the right to use consumer products which do not harm their health and the environment without special precautionary efforts.

## Conclusions

The consumer’s ‘SVHC right to know’ is one of the elements in the REACH Regulation that should lead to the gradual substitution of SVHCs in consumer articles, but it is not designed to be a comfortable and popular way of information for consumers.

This survey is the first that analysed in detail the awareness, acceptance, and evaluation of the ‘SVHC right to know’ in consumers with high motivation and interest in chemicals in everyday products. The results show that only a minority of these ‘best-case’ consumers are familiar with the ‘SVHC right to know’ and even less make SVHC inquiries. This means that presently consumer requests cannot be regarded as effective stimulus for suppliers to eliminate SVHCs from their articles. Improvements are needed and worth new efforts, because the ‘SVHC right to know’ is a very promising tool: it increases the awareness on SVHC in articles, it enables consumers to have greater access to the information on chemicals in articles, and it strengthens their market power. The results of this study also reveal that consumers need much more support to understand the SVHC information which could enable them to make informed purchase decisions and to take appropriate action in their daily routine. Furthermore, improvements are needed that take consumers more seriously as important stakeholders who could make this communication instrument work and who should in the end profit from the SVHC provisions on the way to the non-toxic environment.

The focus of this survey on consumer aspects of the ‘SVHC right to know’ should not divert from the urgency to reduce the global use of resources as pivotal sustainability strategy. Most participants in this survey were of the opinion that consumers carry a large part of the responsibility for the reduced use of substances harmful for the human health and the environment (Question 14) [10], and they are right: it would be a great success in the process of sustainable development if a majority of consumers took their responsibility serious and reduced their personal consumption.

### Additional files

**Additional file 1: Figure S1.** Age groups of study participants and self-reported knowledge in chemistry. Diagram shows the proportions of female (left) and male (right) study participants who have (very) good self-reported chemical knowledge.

**Additional file 2: Figure S2.** Flowchart of the questionnaire with number of participants. Numbers decline from 1321 to 1030 as 291 participants ceased answering the questionnaire before the end.

**Additional file 3.** Original German version of the survey.

## Appendix: Text of the survey (Questions 1 and 15–38)

Welcome!

Welcome to our survey on **chemicals which are harmful for human health or the environment and used in everyday products** (such as furniture, electronic appliances, and plastic products). Our aim is to make suggestions for improving the legal framework. Your answers are valuable contributions for our work. We would be happy if you could answer the questionnaire and forward it to friends, so that we will receive a large number of answers in the short time span. The analysis of the answers will be anonymous. In case **you are interested in the results of our study**, please send an e-mail to the address indicated at the end of the questionnaire and we will send them to you. We will cast lots among these addresses and give away three **vouchers** worth 100 Euros each to be redeemed at the shop of the BUND (friends of the earth Germany) (http://www.bundladen.de).

Prof. Dr. Ursula Klaschka (**University of Applied Sciences Ulm**) is responsible for the survey, which will be conducted until 31 October 2016.

Questions with circular symbols allow only a single answer, whereas questions with rectangular symbols allow several answers.

Thank you for your participation!

Do you want to know more? Here are the **essentials in brief**.

Lots of everyday products contain **harmful substances**. The **hazard pictograms** and safety phrases on the containers of paints, varnishes, washing, and cleansing products depict the dangers of such a mixture of chemicals (http://www.umweltbundesamt.de/themen/chemikalien/einstufung-kennzeichnung-von-chemikalien). There are also recommendations for safe handling on the container which help you to reduce your personal risk when dealing with the product and to avoid damage to the environment. Ingredients are listed on the containers/packaging of personal care products and washing and cleansing products. In contrast to that, there is no legal obligation that other articles such as electronic appliances, plastic articles, or furniture have to be labelled with regard to their content of substances harmful to human health or the environment.

You have the right to ask manufacturers of objects, such as electronic appliances, plastic products, or furniture, whether ‘substances of very high concern’ are present in a specific product above a certain concentration (**SVHC right to know**). These substances are, for example, carcinogenic, mutagenic, toxic for reproduction, or very critical for the environment. Examples are certain plasticizers for plastic materials (phthalates), cadmium compounds, or arsenic compounds. A list of these so-called ‘substances of very high concern” is compiled in the framework of the European Chemical Regulation REACH (*Registration, Evaluation, and Authorisation of Chemicals*). This list is extended regularly and available on the Internet under, for example, [http://www.reach-clp-biozid-helpdesk.de/de/REACH/Kandidatenliste/Kandidatenliste.html]. It needs not to be written on an article whether it contains these substances. Instead, you may send an inquiry for a certain article to the manufacturer or the distributor which he must answer within 45 days, in case such a substance is present in the article above the threshold of 0.1 wt%. You find a sample letter (http://www.reach-info.de/auskunftsrecht.htm) and an online form (http://www.reach-info.de/verbraucheranfrage.htm) on the homepage of the German Environment Agency (UBA). UBA is currently working on a smartphone app ‘Scan4Chem’ which will facilitate the information request. The app ToxFox by the BUND (Friends of the Earth Germany) will consider substances of very high concern in addition to endocrine substances in future.

One of the aims of the **right to know** by the European Regulation on chemicals (REACH) is that manufacturers replace these substances by less harmful substances in the long term. Please find further information under (http://www.reach-info.de/svhc.htm) or (http://www.reach-clp-biozid-helpdesk.de/de/REACH/SVHC-Roadmap/Roadmap.html).
