# Supplementary figures and images for: Do consumers care about substances of very high concern in articles?
Source: Environ Sci Eur. 2018 Aug 21;30(1):29. doi: 10.1186/s12302-018-0153-1 (PMC6105235; doi:10.1186/s12302-018-0153-1)

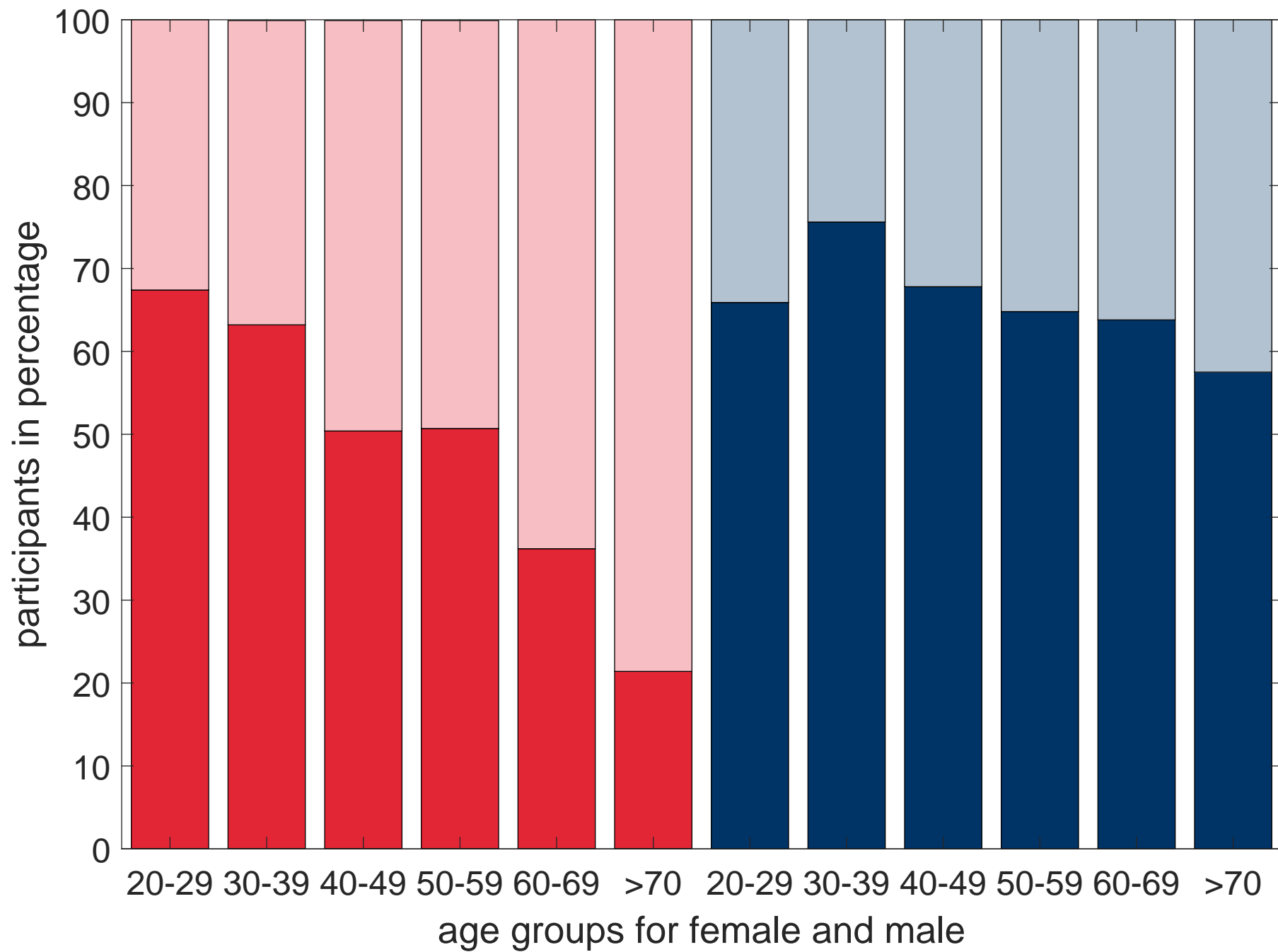

Supplement: Supplementary file 1 — Additional file 1: Figure S1. Age groups of study participants and self-reported knowledge in chemistry. Diagram shows the proportions of female (left) and male (right) study participants who have (very) good self-reported chemical knowledge. [file 12302_2018_153_MOESM1_ESM.pdf]

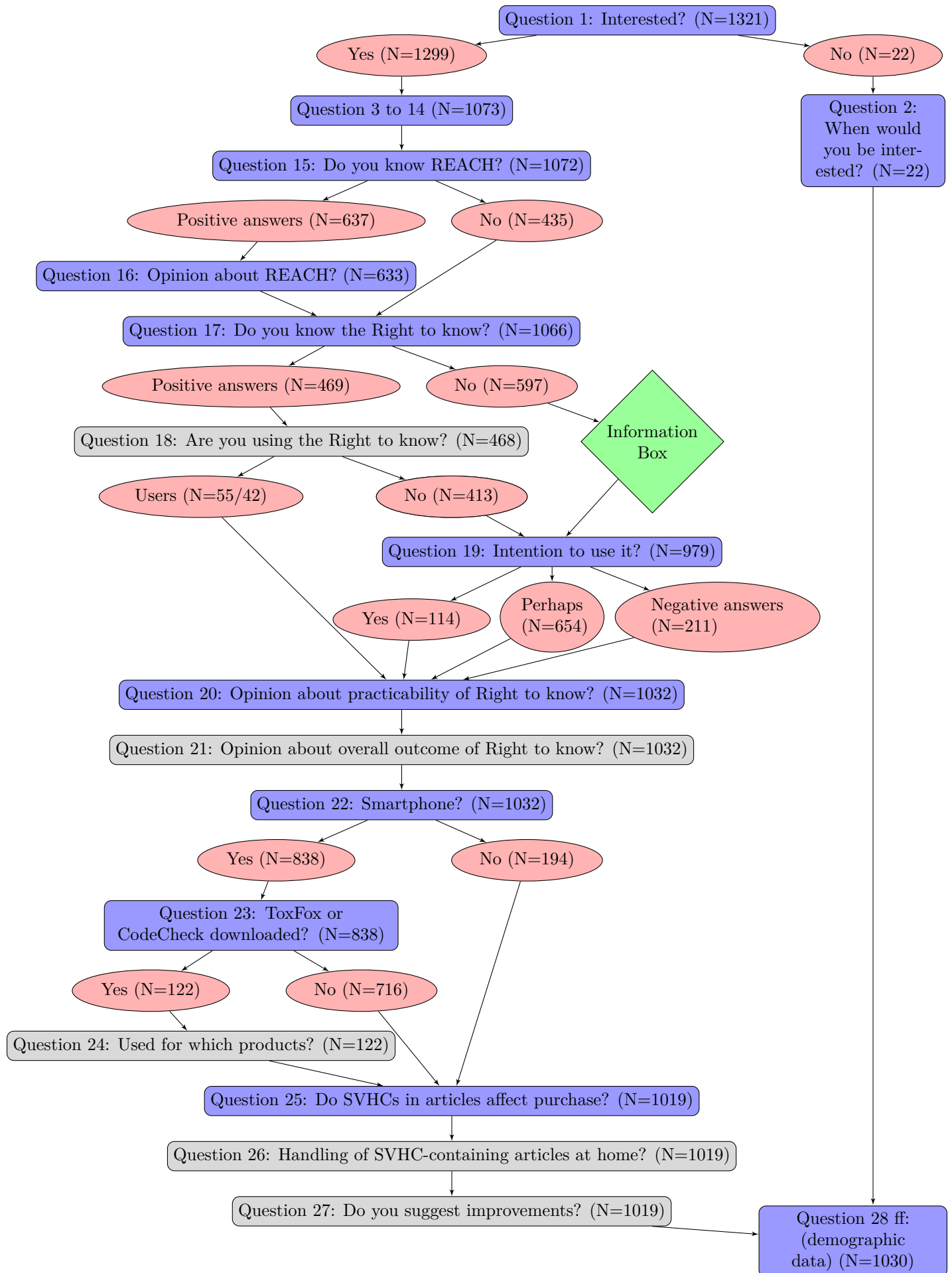

Supplement: Supplementary file 2 — Additional file 2: Figure S2. Flowchart of the questionnaire with number of participants. Numbers decline from 1321 to 1030 as 291 participants ceased answering the questionnaire before the end. [file 12302_2018_153_MOESM2_ESM.pdf]
