# Supplementary material for: Do consumers care about substances of very high concern in articles?
Source: Environ Sci Eur. 2018 Aug 21;30(1):29. doi: 10.1186/s12302-018-0153-1 (PMC6105235; doi:10.1186/s12302-018-0153-1)
Supplement: Supplementary file 3 — Additional file 3. Original German version of the survey. [file 12302_2018_153_MOESM3_ESM.docx]

Herzlich willkommen zu unserer Umfrage zum Thema **gesundheits- und umweltgefährdende Chemikalien in Alltagsprodukten** (wie z.B. Möbel, Elektronikgeräte, Kunststoffprodukte). Wir möchten Vorschläge zur Verbesserung der europäischen Regelungen erarbeiten, und dazu können Sie mit Ihren Antworten einen wichtigen Beitrag leisten. Wir würden uns freuen, wenn Sie diesen Fragebogen beantworten und wenn möglich an Freunde und Bekannte **weiterleiten** könnten, damit wir in der kurzen Zeit möglichst viele Antworten erhalten. Die Auswertung erfolgt anonym. Falls Sie **Interesse an den Ergebnissen der Studie** haben, schicken Sie bitte am Ende der Umfrage eine Mail an die dort angegeben Adresse, dann schicken wir sie Ihnen gerne zu. Unter diesen Adressen werden drei **Geldgutscheine** im Werte von je 100 Euro für den BUND-Laden ([www.bundladen.de](http://www.bundladen.de)) ausgelost

Die Umfrage wird unter Federführung von Prof. Dr. Ursula Klaschka (**Hochschule Ulm) in Kooperation mit dem Umweltbundesamt** bis zum 31.10.2016 durchgeführt.

Im Folgenden ist bei Fragen mit kreisförmigen Symbolen nur eine Antwort möglich, während rechteckige Symbole eine Mehrfachauswahl erlauben.

Wir danken für Ihre Teilnahme.

1. Interessieren Sie sich dafür, ob Alltagsprodukte, die Sie verwenden, gesundheits- und umweltgefährdende Chemikalien enthalten?

- Ja, grundsätzlich immer =>3.-15.
- Es interessiert mich nur bei manchen Produkten => 3.-15.
- Nein. =>2. Dann 28.

(Frage 2.-14. s. Hartmann and Klaschka 2017)

15. Kennen Sie die Europäische Chemikalienverordnung **REACH**?

- Ja, recht gut => 16.
- Ja, schon einmal gehört => 16.
- Nein, noch nie gehört => 17.

16. Wie schätzen Sie die REACH-Verordnung ein?

- Ist ein großer Fortschritt für einen besseren Umgang mit Chemikalien
- Ist ein großer Aufwand mit geringem Erfolg
- Ist mir zu kompliziert
- Kann ich nicht beurteilen.
- Anderes:

=>17.

17. Wissen Sie, dass Sie nach REACH das Recht haben, Auskunft über besonders besorgniserregende Stoffe in Alltagsprodukten zu bekommen? (**REACH-Auskunftsrecht**)

- Ja, ich bin darüber recht gut informiert. => 18
- Ja, schon einmal gehört => 18.
- Nein, noch nie gehört => Infobox 1 dann 19.

18. **Nutzen** Sie das REACH-Auskunftsrecht für Verbraucher?

- Nein => 19.
- Ja, ich nutze das Online-Formular des Umweltbundesamtes ([www.reach-info.de/verbraucheranfrage.htm](http://www.reach-info.de/verbraucheranfrage.htm)). => 20.
- Ja, ich nutze den Musterbrief des Umweltbundesamtes ([www.reach-info.de/auskunftsrecht.htm](http://www.reach-info.de/auskunftsrecht.htm)). => 20
- Ja, ich nutze Anfragemöglichkeiten per App (z.B. ToxFox ([www.bund.net/toxfox)](http://www.bund.net/toxfox)).
- Ja, ich formuliere selbst meine Anfragen an die Hersteller und schicke sie per E-Mail oder Briefpost ab. => 20.

Infobox 1: Sie haben gemäß REACH das Recht, zu erfahren, ob in einem Produkt im Handel sogenannte „besonders besorgniserregende Stoffe“ enthalten sind. Diese sind z.B. krebserzeugend, erbgutverändernd, fortpflanzungsschädigend oder besonders kritisch für die Umwelt, weil sie langlebig sind und sich in Organismen anreichern. Es steht nicht auf dem Produkt, ob diese Stoffe enthalten sind. Sie können stattdessen eine **Anfrage für das jeweilige Produkt an den Hersteller oder Lieferanten** schicken, auf die dieser innerhalb von 45 Tagen antworten muss, wenn solch ein Stoff im Produkt über 0,1 Gewichts-% enthalten ist. Auf der Homepage des Umweltbundesamtes gibt es dazu einen Musterbrief und ein Online-Formular ([www.reach-info.de/auskunftsrecht.htm](http://www.reach-info.de/auskunftsrecht.htm)). Das Umweltbundesamt erarbeitet derzeit eine App „**Scan4Chem**“, die die Abfragen noch mehr erleichtern wird. Auch die App des BUND (Bund für Umwelt und Naturschutz) **Toxfox** wird in Zukunft neben den hormonartig wirkenden Stoffen auch die besonders besorgniserregenden Stoffe berücksichtigen.

Zu beachten ist, dass sich das REACH Auskunftsrecht vor allem auf Gegenstände bezieht, nicht auf flüssige und pulverförmige Produkte, die anderweitig geregelt sind (Gefahren-Piktogramme auf z.B. Farben Klebstoffen, Reinigungsmitteln usw. oder Angabe der Inhaltsstoffe auf der Verpackung z.B. bei Körperpflege- und Reinigungsmitteln).

19. Haben Sie vor, das REACH-Auskunftsrecht in Zukunft zu **nutzen**?

- Ja
- Vielleicht
- Nein, ist mir zu kompliziert
- Nein, dafür habe ich keine Zeit
- Nein, daran habe ich kein Interesse
- 20

20. Was **halten** Sie von dem REACH-Auskunftsrecht?

- finde ich gut
- finde ich nicht verbraucherfreundlich. Es ist für mich im Alltag zu aufwändig.
- funktioniert nicht, da Firmen nicht antworten
- finde ich zu kompliziert
- ist mir nicht wichtig
- kann ich nicht beurteilen
- anderes:

=> 21

21. **Wozu führt** Ihrer Meinung nach das REACH-Auskunftsrecht?

- besserer Verbraucherschutz
- besserer Schutz der Umwelt
- geringerer Einsatz besonders besorgniserregender Stoffe durch Hersteller
- großer Verwaltungsaufwand in den Firmen für die korrekte Beantwortung der Verbraucheranfragen
- großer Überwachungsaufwand bei Behörden
- Verunsicherung der Konsument/innen
- kein besserer Verbraucher- und Umweltschutz, weil es zu wenig genutzt wird
- kein besserer Verbraucher- und Umweltschutz, auch wenn es in großem Maße genutzt wird
- zu gar nichts
- kann ich nicht beurteilen
- anderes:

=22.

22. Haben Sie ein **Smartphone**?

- Ja => 23.
- Nein => 25.

23. Haben Sie die Apps **ToxFox** und/oder **CodeCheck** darauf installiert?

- ToxFox => 24.
- CodeCheck => 24.
- ToxFox und CodeCheck => 24
- Keines der beiden => 25.

24. Wofür **verwenden** Sie eine der beiden genannten Apps?

- für Produkte, die Sie kaufen möchten
- für bereits gekaufte Produkte
- für Produkte, die Sie gar nicht kaufen wollen
- gar nicht
- 25.

25. **Kaufen** Sie Produkte/Würden Sie Produkte kaufen, auch wenn laut Firmenantwort besonders besorgniserregende Stoffe darin sind?

- Ja
- Nein
- Es kommt auf das Produkt an
- 26.

26. **Was machen Sie/**Was würden Sie machen, wenn Sie ein Produkt gekauft haben und nachträglich feststellen, dass es besonders besorgniserregende Stoffe enthält?

- Ich verwende das Produkt dennoch.
- Ich informiere mich genauer über den besonders besorgniserregenden Stoff.
- Ich verwende das Produkt seltener und kaufe es beim nächsten Mal nicht mehr.
- Ich verwende das Produkt nicht (mehr).
- Ich werfe es in den Müll.
- Ich sage es weiter.
- Es kommt auf das Produkt an.
- Anderes:……
- 27.

27. Welche **Verbesserungsvorschläge** haben Sie für das REACH-Auskunftsrecht?

- Keine, ich finde es **gut so**.
- Ich **nutze es nicht**, daher habe ich auch **keine Änderungswünsche**.
- Ich möchte **nicht bei jedem einzelnen Produkt** eine Anfrage stellen müssen, sondern die Information einfacher bekommen.
- Ich möchte eine **schnellere Auskunftspflicht**. Eine Frist von 45 Tagen, bis ich eine Antwort bekommen kann, ist mir zu lang.
- Ich möchte **leicht verständliche Informationen** über **alle** gefährliche Stoffe, z.B. auch allergieauslösende Stoffe, in meinen Alltagsprodukten **auf der Produktverpackung**.
- Ich möchte **leicht verständliche Informationen** über **alle** gefährliche Stoffe, z.B. auch allergieauslösende Stoffe, in meinen Alltagsprodukten **in öffentlich zugänglichen Datenbanken**.
- Ich wünsche mir die **Namen** besonders besorgniserregender Stoffe auf der **Produktverpackung**.
- Ich wünsche mir eine Kennzeichnung der Produkte mit einem **Ampelsystem** (Grün = besonders besorgniserregende Stoffe < 0,01%, Gelb = geringe Menge von einem besonders besorgniserregenden Stoff, Rot = größere Menge oder mehrere besonders besorgniserregende Stoffe).
- Ich wünsche mir auf der Produktverpackung ein **Piktogramm**, das anzeigt, ob besonders besorgniserregende Stoffe im Produkt enthalten sind.
- Ich wünsche mir, dass die **Wirkung dieser Stoffe** (z.B. krebserregend, erbgutverändernd, fortpflanzungsschädigend oder besonders umweltgefährdend) auf der Produktverpackung leicht erkennbar ist.
- Ich wünsche mir auf Produkten, die diese Stoffe nicht enthalten, einen **Hinweis**: „Dieses Produkt ist frei von besonders besorgniserregenden Stoffen“.
- Ich wünsche mir Informationen zu diesen Stoffen auf der **Homepage der Hersteller**.
- Ich wünsche mir eine **App**, die aufgrund des Barcodes direkt im Laden anzeigt, ob besonders besorgniserregende Stoffe im Produkt enthalten sind und welche möglichen Wirkungen diese haben.
- Ich wünsche mir **mehr Überwachung** der Produkte durch die zuständigen Behörden.
- Ich wünsche mir, dass von dem Recht, dass Firmen mit einer **Geldbuße** bis zu 50.000 Euro bestraft werden, wenn sie ihrer Auskunftspflicht nicht nachkommen, mehr Gebrauch gemacht wird.
- Ich wünsche mir, dass besonders besorgniserregende Stoffe in Alltagsprodukten möglichst bald **verboten** werden.
- Weiß nicht
- Andere Vorschläge:
- 28.

**Angaben zur Person**

28. Sind Sie männlich oder weiblich?

- Männlich
- weiblich

29. Wie alt sind Sie?

- unter 20
- 20-29
- 30-39
- 40-49
- 50-59
- 60-69
- 70 oder älter

30. Haben Sie Kinder, die unter 18 Jahre alt sind?

- Ja
- nein

31. Was ist Ihr höchster Bildungsabschluss?

- Derzeit Schüler
- Derzeit im Studium oder in der Ausbildung
- abgeschlossene Ausbildung /Lehre
- abgeschlossene Ausbildung zum Meister / Fachwirt
- abgeschlossenes Hochschulstudium/Promotion
- sonstiges

32. Wie schätzen Sie Ihre Kenntnisse von Chemie ein?

- Keine bis geringe
- Gute
- sehr gute

33. Haben Sie beruflich mit Chemikalien und REACH zu tun?

- Ja
- Nein

34. Sind Sie Mitglied in einer Umweltorganisation?

- Ja
- Nein

35. Sind Sie Mitglied in einer Verbraucherschutzorganisation?

- Ja
- Nein

36. Sind Sie EU-Bürger?

- Ja
- Nein

37. Ist bei Ihnen oder in Ihrer Familie eine Unverträglichkeit von Chemikalien bekannt?

- Ja
- Nein

**Sind Sie an den Ergebnissen dieser Umfrage interessiert?** Dann schicken wir sie Ihnen gerne zu. Damit nehmen Sie auch an der Verlosung der drei Gutschein im Wert von **100 Euro** beim **BUND-Laden** ([www.bundladen.de](http://www.bundladen.de)) teil. Um die Anonymität Ihrer Antworten zu gewährleisten, schicken Sie uns dazu bitte eine E-Mail mit dem **Betreff „Verlosung“** an folgende Adresse: **REACH-Umfrage-2016@hs-ulm.d**e.

38. Haben Sie **Kommentare und Anmerkungen**? .......

Wollen Sie mehr zu dem Thema wissen? Hier ist **in Kürze das Wesentliche** zusammengefasst:

In sehr vielen Alltagsprodukten sind **Gefahrstoffe** enthalten. Bei Farben, Lacken, Wasch- und Reinigungsmitteln können Sie anhand der **Gefahrenpiktogramme**

und Sicherheitshinweise auf den Verpackungen schnell erkennen, welche Gefahren von der Stoffmischung ausgehen ([www.umweltbundesamt.de/themen/chemikalien/einstufung-kennzeichnung-von-chemikalien](http://www.umweltbundesamt.de/themen/chemikalien/einstufung-kennzeichnung-von-chemikalien)). Es sind dort auch Empfehlungen aufgedruckt, wie Sie sich verhalten können, um Ihr persönliches Risiko beim Umgang mit dem Produkt möglichst gering zu halten und die Umwelt nicht zu gefährden. Bei Körperpflege- und Wasch- und Reinigungsmitteln werden die Inhaltsstoffe direkt auf der Verpackung angegeben. Auf anderen Produkten, wie z.B. Elektronikgeräten, Kunststoffartikeln oder Möbeln sind Angaben auf den Verpackungen, ob gesundheits- oder umweltgefährdende Stoffe enthalten sind, nicht vorgeschrieben.

Bei Gegenständen wie Elektronikgeräten, Kunststoffartikeln und Möbeln haben Sie das Recht, beim Hersteller zu erfragen, ob sogenannte besonders besorgniserregende Stoffe über einer bestimmten Menge enthalten sind („**REACH-Auskunftsrecht**“). Diese Stoffe sind z.B. krebserzeugend, erbgutverändernd, fortpflanzungsschädigend oder besonders gefährlich für die Umwelt. Beispiele sind bestimmte Weichmacher für Kunststoffe (Phthalate), Cadmiumverbindungen oder Arsenverbindungen. Im Rahmen der europäischen Chemikaliengesetzgebung (REACH-Verordnung, *Registration, Evaluation and Authorization of Chemicals*) wird eine Liste dieser sogenannten „besonders besorgniserregenden Stoffe“ erstellt. Diese Liste wird regelmäßig erweitert und ist im Internet z.B. unter [http://www.reach-clp-biozid-helpdesk.de/de/REACH/Kandidatenliste/Kandidatenliste.html] zu finden. Bei Gegenständen muss nicht auf dem Produkt angegeben werden, ob diese Stoffe enthalten sind. Sie haben aber das Recht, eine Anfrage für das jeweilige Produkt an den Lieferanten oder Hersteller des Produkts zu schicken. Dieser muss innerhalb von 45 Tagen antworten, wenn ein besonders besorgniserregender Stoff im Produkt über 0,1 Gewichts-% enthalten ist. Auf der Homepage des Umweltbundesamtes gibt es dazu einen **Musterbrief** und ein **Online-Formular** ([www.reach-info.de/auskunftsrecht.htm](http://www.reach-info.de/auskunftsrecht.htm)). Unter der Federführung des Umweltbundesamtes ist eine App „**Scan4Chem**“ in Arbeit, die Abfragen noch weiter erleichtern wird. Auch die App des BUND (Bund für Umwelt und Naturschutz) **ToxFox** wird in Zukunft neben den hormonartig wirkenden Stoffen auch die besonders besorgniserregenden Stoffe berücksichtigen.

Die europäische Verordnung zu Chemikalien (REACH) verfolgt mit dem Auskunftsrecht unter anderem das Ziel, dass Hersteller diese Stoffe langfristig durch weniger gefährliche ersetzen. Weitere Infos unter ([www.reach-info.de/svhc.htm](http://www.reach-info.de/svhc.htm)) oder ([www.reach-clp-biozid-helpdesk.de/de/REACH/SVHC-Roadmap/Roadmap.html](http://www.reach-clp-biozid-helpdesk.de/de/REACH/SVHC-Roadmap/Roadmap.html) ).
